# Ion Channel Expression in the Developing Enteric Nervous System

**DOI:** 10.1371/journal.pone.0123436

**Published:** 2015-03-23

**Authors:** Caroline S. Hirst, Jaime P. P. Foong, Lincon A. Stamp, Emily Fegan, Stephan Dent, Edward C. Cooper, Alan E. Lomax, Colin R. Anderson, Joel C. Bornstein, Heather M. Young, Sonja J. McKeown

**Affiliations:** 1 Department of Anatomy & Neuroscience, University of Melbourne, Parkville, Victoria, Australia; 2 Department of Physiology, University of Melbourne, Parkville, Victoria, Australia; 3 Department of Molecular & Human Genetics, Baylor College of Medicine, Houston, Texas, United States of America; 4 Department of Biomedical and Molecular Sciences, Queen's University, Kingston, Ontario, Canada; 5 Department of Medicine, Queen's University, Kingston, Ontario, Canada; Laboratoire de Biologie du Développement de Villefranche-sur-Mer, FRANCE

## Abstract

The enteric nervous system arises from neural crest-derived cells (ENCCs) that migrate caudally along the embryonic gut. The expression of ion channels by ENCCs in embryonic mice was investigated using a PCR-based array, RT-PCR and immunohistochemistry. Many ion channels, including chloride, calcium, potassium and sodium channels were already expressed by ENCCs at E11.5. There was an increase in the expression of numerous ion channel genes between E11.5 and E14.5, which coincides with ENCC migration and the first extension of neurites by enteric neurons. Previous studies have shown that a variety of ion channels regulates neurite extension and migration of many cell types. Pharmacological inhibition of a range of chloride or calcium channels had no effect on ENCC migration in cultured explants or neuritogenesis *in vitro*. The non-selective potassium channel inhibitors, TEA and 4-AP, retarded ENCC migration and neuritogenesis, but only at concentrations that also resulted in cell death. In summary, a large range of ion channels is expressed while ENCCs are colonizing the gut, but we found no evidence that ENCC migration or neuritogenesis requires chloride, calcium or potassium channel activity. Many of the ion channels are likely to be involved in the development of electrical excitability of enteric neurons.

## Introduction

The enteric nervous system (ENS) develops from neural crest-derived cells, the majority of which migrate from the vagal region of the neural tube [1,2]. These enteric neural crest-derived cells (ENCCs) migrate rostro-caudally along the gut beginning around embryonic day (E) 9.5 in mice, and do not reach the anal end of the gut until E14.5 [3–5]. During their migration, a sub-population of ENCCs starts to express neuronal markers [6–9]. Furthermore, many neurons begin to extend caudally-projecting neurites that grow in close association with migrating ENCCs [10–13]. Many transcription factors, receptors, extracellular ligands and adhesion molecules that regulate ENCC migration and differentiation have been identified [5,14–16].

A growing body of evidence indicates that ion channels regulate cell motility, neurite outgrowth and axon guidance in a variety of cell types [17–19]. Ion channels regulate cell migration by mediating changes in cell shape and volume through osmotic activity, and by interactions with components of the cell migration machinery including the cytoskeleton and integrins [19–22]. For example, glioma cell migration requires activity of K^+^ and Cl^-^ channels [20,23–26], platelet migration requires activity of Ca^2+^ and K^+^ channels [27], neuroblast migration along the rostral migratory stream involves K^+^ channels [28], and the migration of post-mitotic granule cells in the cerebellum [29] and trunk neural crest cells in the salamander [30] are regulated by Ca^2+^ channels. In growing axons, ion channels can regulate the rate of axon growth, the response to different guidance cues and target recognition. For example, voltage-dependent K^+^ channels have been reported to regulate the rate of growth and guidance of retinal ganglion cell axons [31], activity mediated by voltage-dependent Ca^2+^ channels regulates transcription of receptors for axon guidance molecules, which in turn regulates the growth rate of the neurites [32], Cl^-^ channels are involved in nerve growth factor-induced neurite outgrowth in PC12 cells and cortical neurons [33] and Ca^2+^ channels can interact with laminin to inhibit neurite growth after arrival at the target tissue [34].

We have previously shown the expression of several sodium channels in the ENCCs at E11.5, namely Na_v_1.3, Na_v_1.5, Na_v_1.6, Na_v_1.7 and Na_v_1.9 [35]. Other studies have detected expression of the calcium channel genes *Cacng4* [36] and *Cacng2* [37] and the chloride channel *Clcn4-2* [36] in E14.5 ENCC. However, there has been no comprehensive study of the expression of ion channels by ENCCs, and little is known about whether ion channels play roles in ENCC migration and/or neurite formation during ENS development. Therefore, we first investigated the expression of ion channels by ENCCs using a PCR-based array. We found that many ion channels, including Cl^-^, Ca^2+^, K^+^ and Na^+^ channels are already expressed by ENCCs at E11.5, and there is an increase in the expression of numerous ion channel genes between E11.5 and E14.5. As this time period coincides with population of the gut by ENCCs and the first extension of neurites by enteric neurons, we then examined the effects of pharmacological inhibition of many of the ion channels on ENCC migration and neurite formation. None of the Ca^2+^ or Cl^-^ blockers examined had significant effects on migration or neurite formation. The non-selective K^+^ channel blockers, TEA and 4-AP, retarded ENCC migration and inhibited neurite formation, but only at concentrations that also resulted in significant cell death.

## Methods

### Animals

Wild-type and *Ednrb-hKikGR* mice [11], both on a C57Bl/6 background, were used. All ENCCs in *Ednrb-hKikGR* mice express the fluorescent protein, KikGR [11]. Mice were bred in the Biomedical Animal Facility at the University of Melbourne, and were SPF status (free from common mouse viruses/bacteria and parasites). They were housed at 3–5 mice/cage in Tecniplast individually ventilated cages (Green line) with Fybrecycle paper bedding (autoclaved prior to use) and maintained on a 12/12 light/dark cycle at 21°C. The entire study was approved by the University of Melbourne Anatomy and Neuroscience, Pathology, Pharmacology and Physiology Animal Ethics Committee (Permit 1312869).

### RNA extraction

Enteric neural crest cells were FACS sorted from freshly dissociated E11.5 and E14.5 *Ednrb-hKikGR* mice as described previously [38], between 10 AM – 2PM. FACS sorted cells were collected in phosphate buffered saline (PBS), pelleted, excess PBS removed and immediately frozen at -80°C. The small intestine was isolated from postnatal day (P)0 and adult mice in sterile DMEM/F12, and the mucosa removed with forceps, between 9 AM—3 PM. The remaining muscle, myenteric plexus and serosa were immediately transferred into 1ml of RNAlater (Qiagen).

Total RNA was extracted from approximately 1x10^6^ freshly dissociated and purified E11.5 and E14.5 FACS-sorted ENCCs using Qiashredder and RNeasy mini kit (Qiagen), including the on-column DNase treatment, according to manufacturer’s instructions. Total RNA was extracted from P0 and adult gut using Trizol (Life Technologies Invitrogen), then purified further using RNeasy mini columns and on-column DNase treatment (Qiagen), according to manufacturer’s instructions. RNA quality and quantity were tested by spectrophotometry using a NanoDrop 1000 and electrophoresis, and only RNA meeting the criteria detailed by SABiosciences RT^2^ Profiler PCR Array System was used in the arrays.

### PCR array

0.2 μg of total RNA was converted to cDNA for each age, using the RT^2^ First Strand kit (SA Biosciences). Real time PCR was performed on a 384 well RT^2^ Profiler PCR array for Mouse Neuroscience Ion channels and Transporters (PAMM-036, 2011, SA Biosciences) using SA Biosciences RT^2^ qPCR Master Mix, and run on an ABI 7900HT Real time instrument. Three separate PCRs were performed, in which cDNA from each age was loaded onto 96 wells of the 384 well PCR plate. Real time PCR was run and analysed according to SA Biosciences recommended protocols, and data analysed using the SA Biosciences web portal data analysis.

### Reverse transcription-polymerase chain reaction (RT-PCR)

RNA was extracted from E14.5 freshly dissociated and purified ENCCs, and from adult whole brain as described above. The concentration of total RNA in each sample was measured using a NanoDrop ND-1000 spectrophotometer. cDNA was synthesised using the iScript Advanced cDNA Synthesis Kit for RT-qPCR (Bio-Rad); 100-350ng of total RNA was used in a final reaction volume of 20 μl according to the manufacturer’s instructions. Control reactions using no reverse transcriptase or substituting cDNA with water were run in parallel for each tissue.

RT-PCR was conducted using intron-spanning specific primer pairs (S1 Table) and a touchdown PCR (TD-PCR) cycling program (S2 Table). A standard RT-PCR protocol was used consisting of MangoTaq (0.2 μl, Bioline), dNTP Mix (0.8 mM, Bioline), MgCl_2_ (2 mM, Bioline), and primer pairs (1 μM each) in a final reaction volume of 20 μl. RT-PCR products (5–15 μl) were resolved by gel electrophoresis on a 1–2% agarose gel, containing either ethidium bromide (0.06 μg, Sigma) or GelRed (0.5x, Biotium), together with 100 bp DNA Ladder (Life Technologies) or Ready-to-Use 100 bp DNA Ladder (Biotium) to estimate product size. Control reactions were run in parallel for each RT-PCR reaction.

### Drugs

The following drugs were used: bumetanide (200 μM, Sigma-Aldrich, Castle Hill, NSW, Australia), 5-Nitro-2-(3-phenylpropylamino) benzoic acid (NPPB, 100 μM, Sigma-Aldrich), ω-agatoxin IVA (200 nM, Alomone labs, Jerusalem, Israel), ω-conotoxin GVIA (5 μM, Alomone labs), mibefradil dihydrochloride (1 μM, Sigma-Aldrich,), nicardipine (2.5 μM, Sigma-Aldrich), tetraethylammonium (TEA, 2 mM, 10 mM or 30 mM, Sigma-Aldrich), 4-aminopyridine (4-AP, 0.1mM or 5 mM, Sigma-Aldrich), BDS-I (2.5 μM, Alomone labs) and linopirdine (10 μM, Sigma-Aldrich). The concentrations of the drugs used were based on previous publications [23,31,39–43]. With the exception of NPBB, BDS-I, TEA and 4-AP, all drugs were prepared as 1000-fold stock solutions of the highest final concentration used, and diluted to their final concentration in tissue culture medium (TCM: DMEM/F12 containing 10% fetal bovine serum, 6 mg/ml penicillin/streptomycin and 20 mM GlutaMAX (all from Invitrogen, Mulgrave, VIC, Australia). TEA was prepared as 80-fold, NPBB and BDS-I as 100-fold, and 4-AP as 400-fold stock solutions of the highest final concentration used.

### Catenary culture

Mid and hindgut from E11.5 mice were dissected and strung across a “V” cut into a piece of filter paper [44]. These catenary cultures were placed into 20 μl of TCM in wells of a Terasaki plate. The explants were grown for 48 h at 37°C in control TCM or TCM with drugs (selected at random), and then processed for immunohistochemistry using the ENCC marker, Sox10 [45], and the neuronal marker, Tuj1 [46] (S3 and S4 Tables). The distance migrated was determined as described previously; in brief the distance between the most caudal Sox10+ cell and the ileocaecal junction was measured using Fiji software [47]. These experiments were set up at 3–5 PM.

### Neurite outgrowth and cell death assays

The small and large intestines were dissected from E14.5 mice, and dissociated as described previously [38]. Following the addition of TCM and gentle trituration, the cell suspension was filtered through a 40 μm cell strainer, and centrifuged for 2 min at 2000 rpm. Cells were resuspended in TCM and 200 μl aliquots of cells were placed as a drop on sterilised 18 mm round coverslips within the wells of a 12-well plate at a density of 4x10^5^ cells/well. The cells were left for 3 h at 37°C to adhere, then the media with any non-attached cells was removed, and replaced with either control TCM or TCM containing drug (selected at random). Cultures were incubated for a further 9 h, and then fixed in 4% paraformaldehyde (PFA) for 15 min before being processed for Tuj1 immunostaining to identify neurons. Images of randomly chosen fields of view were taken using a X10 objective lens, and then the proportions of Tuj1+ cell bodies possessing one or more neurites that were >50% of the diameter of the cell body was counted using the cell counter plug-in on Fiji software. These analyses (imaging and counting) were all done blinded. Some cultures were also grown in the presence of drugs for 21 h prior to fixation. To examine apoptosis, gut from E14.5 *Ednrb-hKikGR* mice was dissociated, the cells allowed to adhere to coverslips for 3 h and then exposed to drugs for 9 h or 21 h prior to fixation and processing for immunohistochemistry using antisera to activated caspase-3 (S3 and S4 Tables). Random fields of view containing KikGR+ ENCCs were imaged and the percentage of KikGR+ cells that was also activated caspase-3+ determined. Data were analysed using t tests or one way ANOVAs, as appropriate. These experiments were set up at 7–9 AM.

### Immunofluorescence

Wholemount preparations of embryonic gut, cultured explants or cultured cells were processed for immunohistochemistry as described previously [38,48] using primary and secondary antisera shown in S3 and S4 Tables.

For Kv7.3 immunolabelling [49], embryonic mouse guts distal to the stomach were dissected quickly, cryopreserved in 20% sucrose and embedded in OCT, which was rapidly frozen in isopentane. Cryostat sections (10 μm) were thaw-mounted onto Superfrost slides and air dried for 3 minutes before being immersed in ice-cold acetone for 10 minutes. Sections were then processed for immunohistochemistry as described above.

## Results

### E11.5 and E14.5 ENCCs express a variety of ion channels

We performed a QPCR array and compared the expression of ion channels and transporters in E11.5 and E14.5 purified ENCCs. Many ion channels were already expressed at E11.5; over 30 were up-regulated more than 3-fold in E14.5 ENCCS compared to E11.5, three were down-regulated (Table 1), and a further 25 ion channels were expressed at similar levels at both E11.5 and E14.5 (less than 3-fold difference in expression; Table 2). The ion channels that were up-regulated were predominately K^+^, Na^+^ and Ca^2+^ channels (Table 1), and those expressed at similar levels at E11.5 and E14.5 included multiple Cl^-^, K^+^ and Ca^2+^ channels and a variety of transporters (Table 2). We then prioritized several Cl^-^, K^+^ and Ca^2+^ channels that showed substantial gene expression changes, either up or down, between E11.5 and E14.5, and/or that had previously been reported to be expressed by or play a role in migrating cells, for further expression and functional analysis (see below). A number of genes encoding Cl^-^ channels were highly expressed at both E11.5 and E14.5, so their functional roles in ENCC migration and neuritogenesis were also examined.

**Table 1:** Ion channel genes differentially expressed by purified (FACS-sorted) E14.5 ENCCs compared to E11.5 ENCCs.

Gene symbol	Gene name	Channel name	Fold change	Accn #
***Genes upregulated at E14*.*5 compared to E11*.*5***				
**Kcnq3**	**Potassium voltage-gated channel, subfamily Q, member 3**	**Kv7.3**	**170.0**	**NM_152923**
^§^Scn5a	Sodium channel, voltage-gated, type V, alpha	Nav1.5	40.6	NM_021544
^§^Scn9a	Sodium channel, voltage-gated, type IX, alpha	Nav1.7	21.1	NM_018852
**Kcnc4**	**Potassium voltage gated channel, Shaw-related subfamily, member 4**	**Kv3.4**	**15.9**	**NM_145922**
**Kcna2**	**Potassium voltage-gated channel, shaker-related subfamily, member 2**	**Kv1.2**	**15.9**	**NM_008417**
Atp1b1	ATPase, Na+/K+ transporting, beta 1 polypeptide		15.3	NM_009721
Accn1	Amiloride-sensitive cation channel 1, neuronal (degenerin)	Asic2	15.1	NM_007384
**Kcnb1**	**Potassium voltage gated channel, Shab-related subfamily, member 1**	**Kv2.1**	**14.3**	**NM_008420**
Kcnh1	Potassium voltage-gated channel, subfamily H (eag-related), member 1	Kv10.1	13.0	NM_010600
Scn1a	Sodium channel, voltage-gated, type I, alpha	Nav1.1	13.0	NM_018733
^§^Scn3a	Sodium channel, voltage-gated, type III, alpha	Nav1.3	12.8	NM_018732
Kcnj3	Potassium inwardly-rectifying channel, subfamily J, member 3	Kir3.1	12.7	NM_008426
**Kcnj6**	**Potassium inwardly-rectifying channel, subfamily J, member 6**	**Kir3.2**	**12.7**	**NM_010606**
**Cacng2**	**Calcium channel, voltage-dependent, gamma subunit 2**		**12.4**	**NM_007583**
**Kcnq2**	**Potassium voltage-gated channel, subfamily Q, member 2**	**Kv7.2**	**11.5**	**NM_010611**
**Cacnb4**	**Calcium channel, voltage-dependent, beta 4 subunit**		**11.1**	**NM_146123**
**Kcnn3**	**Potassium intermediate/small conductance calcium-activated channel, subfamily N, member 3**	**SK3**	**10.2**	**NM_080466**
Cacna1g	Calcium channel, voltage-dependent, T type, alpha 1G subunit	Cav3.1d	9.3	NM_009783
**Cacnb1**	**Calcium channel, voltage-dependent, beta 1 subunit**		**8.7**	**NM_031173**
**Kcnd2**	**Potassium voltage-gated channel, Shal-related family, member 2**	**Kv4.2**	**7.7**	**NM_019697**
**Kcna5**	**Potassium voltage-gated channel, shaker-related subfamily, member 5**	**Kv1.5**	**6.9**	**NM_145983**
**Kcnd3**	**Potassium voltage-gated channel, Shal-related family, member 3**	**Kv4.3**	**6.8**	**NM_019931**
*Kcnc1	Potassium voltage gated channel, Shaw-related subfamily, member 1	Kv3.1	6.4	NM_008421
Slc18a3	Solute carrier family 18 (vesicular monoamine), member 3		6.4	NM_021712
**Kcnn2**	**Potassium intermediate/small conductance calcium-activated channel, subfamily N, member 2**	**SK2**	**6.3**	**NM_080465**
**Kcnq4**	**Potassium voltage-gated channel, subfamily Q, member 4**	**Kv7.4**	**5.3**	**NM_001081142**
**Cacna1b**	**Calcium channel, voltage-dependent, N type, alpha 1B subunit**	**Cav2.2**	**4.7**	**NM_007579**
**Cacna1c**	**Calcium channel, voltage-dependent, L type, alpha 1C subunit**	**Cav1.2**	**4.1**	**NM_009781**
Atp4a	ATPase, H+/K+ exchanging, gastric, alpha polypeptide		4.0	NM_018731
Scn8a	Sodium channel, voltage-gated, type VIII, alpha	Nav1.6	3.7	NM_001077499
Atp6v0b	ATPase, H+ transporting, lysosomal V0 subunit B		3.7	NM_033617
Slc6a9	Solute carrier family 6 (neurotransmitter transporter, glycine), member 9		3.6	NM_008135
**Cacnb3**	**Calcium channel, voltage-dependent, beta 3 subunit**	**CAB3**	**3.3**	**NM_007581**
***Genes downregulated at E14*.*5***				
**Kcna1**	**Potassium voltage-gated channel, shaker-related subfamily, member 1**	**Kv1.1**	**-5.6**	**NM_010595**
**Clcn5**	**Chloride channel 5**	**Clc-5**	**-4.6**	**NM_016691**
Accn2	Amiloride-sensitive cation channel 2, neuronal	Asic1	-3.5	NM_009597

Only genes showing an average cycle length less than 30 at either E11.5 or E14.5 are shown. Expression of some genes was confirmed at E14.5 by RT-PCR (bold)

§Expression was previously detected in the E11.5 gut [35]

* Expression was not confirmed by RT-PCR at E14.5. Expression of other channels was not examined

**Table 2:** Ion channels expressed at similar levels in E11.5 and E14.5 ENCC.

Gene symbol	Gene name	Channel name	2^-ΔCt		Accn #
			E11.5	E14.5	
Accn3	Amiloride-sensitive cation channel 3	Asic3	0.001115	0.001196	NM_183000
Atp1a1	ATPase, Na+/K+ transporting, alpha 1 polypeptide		0.85963	1.15994	NM_144900
Atp1b2	ATPase, Na+/K+ transporting, beta 2 polypeptide		0.024002	0.055539	NM_013415
Atp2a1	ATPase, Ca++ transporting, cardiac muscle, fast twitch 1		0.000565	0.000822	NM_007504
Atp5b	ATP synthase, H+ transporting mitochondrial F1 complex, beta subunit		1.50881	1.542938	NM_016774
Atp6ap1	ATPase, H+ transporting, lysosomal accessory protein 1		0.088185	0.216314	NM_018794
**Cacna1a**	**Calcium channel, voltage-dependent, P/Q type, alpha 1A subunit**	**Cav2.1**	**0.00776**	**0.02082**	**NM_007578**
**Cacna1h**	**Calcium channel, voltage-dependent, T type, alpha 1H subunit**	**Cav3.2**	**0.135906**	**0.145067**	**NM_021415**
**Clcn2**	**Chloride channel 2**	**Clc-2**	**0.014435**	**0.037717**	**NM_009900**
**Clcn3**	**Chloride channel 3**	**Clc-3**	**0.057997**	**0.131913**	**NM_007711**
**Clcn4-2**	**Chloride channel 4–2**	**Clc4-2**	**0.057945**	**0.151488**	**NM_011334**
**Clcn6**	**Chloride channel 6**	**Clc-6**	**0.004314**	**0.010636**	**NM_011929**
**Clcn7**	**Chloride channel 7**	**Clc-7**	**0.018078**	**0.01939**	**NM_011930**
Clic1	Chloride intracellular channel 1		0.368632	0.421613	NM_033444
Kcna3	Potassium voltage-gated channel, shaker-related subfamily, member 3	Kv1.3	0.004298	0.005646	NM_008418
**Kcna4**	**Potassium voltage-gated channel, shaker-related subfamily, member 4**	**Kv1.4**	**0.000608**	**0.001427**	**NM_021275**
**Kcna6**	**Potassium voltage-gated channel, shaker-related, subfamily, member 6**	**Kv1.6**	**0.007826**	**0.014515**	**NM_013568**
Kcnc3	Potassium voltage gated channel, Shaw-related subfamily, member 3	Kv3.3	0.00548	0.0107	NM_008422
**Kcnf1**	**Potassium voltage-gated channel, subfamily F, member 1**	**Kv5.1**	**0.002713**	**0.004886**	**NM_201531**
Kcnh2	Potassium voltage-gated channel, subfamily H (eag-related), member 2	Kv11.1/ERG1	0.002434	0.005913	NM_013569
**Kcnmb1**	**Potassium large conductance calcium-activated channel, subfamily M, beta member 1**	**Slo-beta**	**0.000842**	**0.000541**	**NM_031169**
**Kcnn1**	**Potassium intermediate/small conductance calcium-activated channel, subfamily N, member 1**	**SK1**	**0.002357**	**0.005262**	**NM_032397**
**Kcnn4**	**Potassium intermediate/small conductance calcium-activated channel, subfamily N, member 4**	**SK4**	**0.000535**	**0.001085**	**NM_008433**
*Kcns1	K+ voltage-gated channel, subfamily S, 1	Kv9.1	0.000794	0.000858	NM_008435
Scn1b	Sodium channel, voltage-gated, type I, beta		0.001173	0.001607	NM_011322
Vdac1	Voltage-dependent anion channel 1		0.356825	0.468966	NM_011694

Only genes showing an average cycle length less than 30 at either E11.5 or E14.5 are shown. Expression of some genes was confirmed at E14.5 by RT-PCR (bold); expression of KCa3.1 (also called IK1 or IK_Ca_), which is encoded by *Kcnn4*, by ENCCs was also previously reported using immunohistochemistry [50].

* Expression was not confirmed by RT-PCR at E14.5. Expression of other channels was not examined.

Gene expression levels were also examined in the P0 and adult gut but could not be quantitatively compared to E11.5 or E14.5 ENCCs, because myenteric plexus with attached external muscle layers and serosa was used for P0 and adult gut for technical reasons. Nonetheless, most genes that were expressed in P0 and adult tissue were also expressed at both E11.5 and E14.5 (S5 Table).

### Inhibition of Cl^-^ channels does not affect ENCC migration or neurite outgrowth

**Fig 1:**
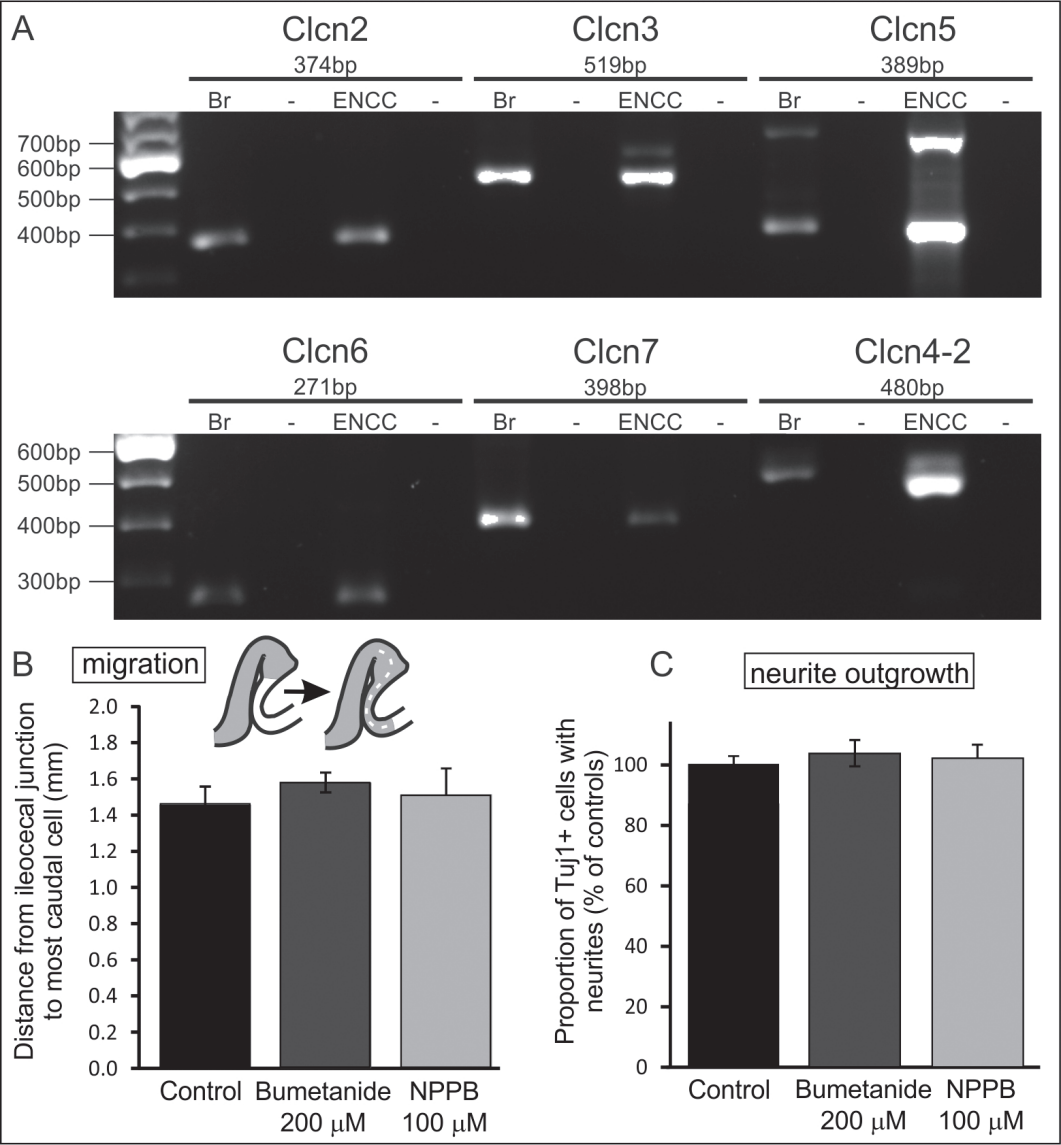
Expression of chloride channels and the effects of chloride channel blockers on ENCC migration and neurite outgrowth. **A.** RT-PCR confirming expression of transcripts encoding 6 chloride channels in FACS-sorted ENCCs from E14.5 gut. Adult mouse brain (Br) was used as a positive control, and-RT was a negative control (-). **B.** Migration assay to assess effects of blocking chloride channels on ENNC migration. Explants of gut were removed from E11.5 mice, when the wavefront of ENCCs (grey) is just beyond the caecum, and grown in culture for 48 hours, during which time the ENCCs migrate into the distal hindgut. The preparations were fixed and the distance from the ileo-caecal junction to the most distal ENCC was then measured (dotted line). There was no significant difference in the distance migrated by ENCCs in explants grown in the presence of bumetanide (n = 9) or NPPB (n = 8) compared to controls (n = 10) (mean ± SEM; one way ANOVA). **C.** Effects of chloride channel blockers on neurite outgrowth. The small and large intestines from E14.5 mice were dissociated, allowed to adhere to coverslips for 3 hours and then exposed to bumetanide or NPPB for 9 hours. The cells were then fixed and processed for immunohistochemistry using an antibody to Tuj1. There was no significant difference in the percentage of Tuj1+ cells that extended neurites between control and drug-treated cultures (one way ANOVA; a minimum of 1750 Tuj1+ cells was examined from 6 coverslips from 2 experiments).

A number of chloride channels were highly expressed at both E11.5 and E14.5 (Table 2). We verified the expression of most of these channels in FACS-sorted ENCCs using RT-PCR (Fig 1A). Inhibition of Cl^-^ channels impairs the ability of glioma cells to migrate by perturbing their ability to make the necessary changes in shape and volume required to migrate through small spaces [23,26,51]. As migrating ENCCs undergo changes in shape to move through the extracellular space between mesenchymal cells and to maintain chain formation [13], we assessed the effect of chloride channel blockers on the migration of ENCC along intact explants of E11.5 gut. NPPB is a non-selective Cl^-^ channel blocker, while bumetanide blocks the activity of NKCC1 and NKCC2 cotransporters, which play a major role in Cl^-^ accumulation [23,52]. Neither NPPB nor bumetanide had a significant effect on the rate of migration of ENCC in cultured explants of gut cultured for 48 h (Fig 1B).

Neuritogenesis was assessed by dissociating the small and large intestines of E14.5 mice; after 3 h to allow cells to adhere to the coverslips, the cells were exposed to bumetanide or NPPB for 9 hours, then fixed and processed for immunohistochemistry using an antibody to Tuj1 to identify neurons. Blocking Cl^-^ channels did not affect the proportion of Tuj1+ cells extending neurites in dissociated cultures of E14.5 gut (Fig 1C).

### Inhibition of Ca^2+^ channels does not affect ENCC migration or neurite outgrowth

**Fig 2:**
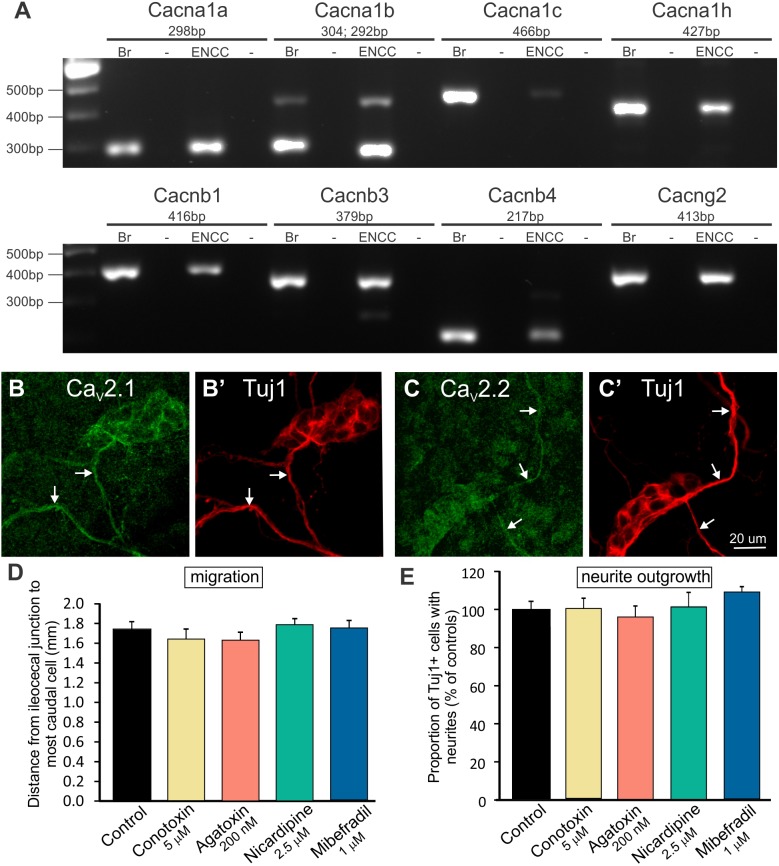
Expression of Ca^2+^ channels and the lack of effect of Ca^2+^ channel blockers on ENCC migration and neuritogenesis. **A.** RT-PCR confirming expression of transcripts encoding 8 calcium channels in purified (FACS-sorted) ENCCs from E11.5 gut. Adult mouse brain (Br) was used as a positive control and-RT was a negative control (-). **B, C.** Immunohistochemistry using antisera to Ca_v_2.1 (**B**) and Ca_v_2.2 (**C**) of dissociated E14.5 gut cultured for 48 hours revealed Ca_v_2.1 and Ca_v_2.2 immunostaining of Tuj1+ neurites (*arrows*). Tuj1+ cell bodies show some Ca_v_2.1 staining, but little, if any, Ca_v_2.2 immunostaining. **D.** There was no significant difference in the distance migrated by ENCCs in explants grown in the presence of the N-type blocker, ω-conotoxin GVIA (n = 9), the P/Q-type blocker, ω-agatoxin IVA (n = 10), the L-type blocker, nicardipine (n = 9) or the T-type blocker, mibefradil (n = 10) compared to controls (n = 11) (mean ± SEM; one way ANOVA; minimum of 2 experiments). **E.** Effects of calcium channel blockers on neuritogenesis. There was no significant difference in the percentage of Tuj1+ cells that extended neurites between control and drug-treated cultures of E14.5 dissociated gut (for controls and each drug, a minimum of 950 Tuj1+ cells was examined from 5 or 6 coverslips from 2 experiments).

The array showed that some Ca^2+^ channels were expressed at similar levels by E11.5 and E14.5 ENCCs (Table 2), while others increased expression between E11.5 and E14.5 (Table 1). Of particular interest was the Ca_v_2.2 subunit of the N-type Ca^2+^ channel, which is involved in neuronal migration and axon guidance in some parts of the nervous system [29,34]. Moreover, we have previously shown that E11.5 enteric neurons respond to electrical field stimulation with Ca^2+^ transients that can be blocked by the N-type Ca^2+^ channel blocker, ω-conotoxin GVIA [39]. *Cacna1b*, which encodes Ca_v_2.2, was upregulated 4.7 fold between E11.5 and E14.5 (Table 1). Other Ca^2+^ channels were also expressed at E11.5 and E14.5, including *Cacna1a*, which encodes the P/Q channel Ca_v_2.1 (Table 2). Expression of most of the Ca^2+^ channels was confirmed by RT-PCR of sorted ENCCs (Fig 2A). Immunohistochemical studies showed expression of Ca_v_2.1 (Fig 2B and 2B’) and Ca_v_2.2 (Fig 2C and 2C’) by neurites of cultured E14.5 enteric neurons.

Pharmacological inhibition of N-, P-, L- and T-type Ca^2+^ channels using ω-conotoxin GVIA (5 μM), ω-agatoxin IVA (200 nM), nicardipine (2.5 μM) and mibefradil (1 μM) respectively, did not affect ENCC migration in E11.5 gut explants (Fig 2D), nor did it affect neuritogenesis of dissociated, cultured E14.5 gut cells (Fig 2E). As the IC_50_ for ω-agatoxin IVA acting at Q-type Ca^2+^ channels is 120 nM [53], it is also unlikely that Q-type channels are involved in neuritogenesis or ENCC migration.

### Effects of inhibition of potassium channels on ENCC migration and neurite outgrowth

**Fig 3:**
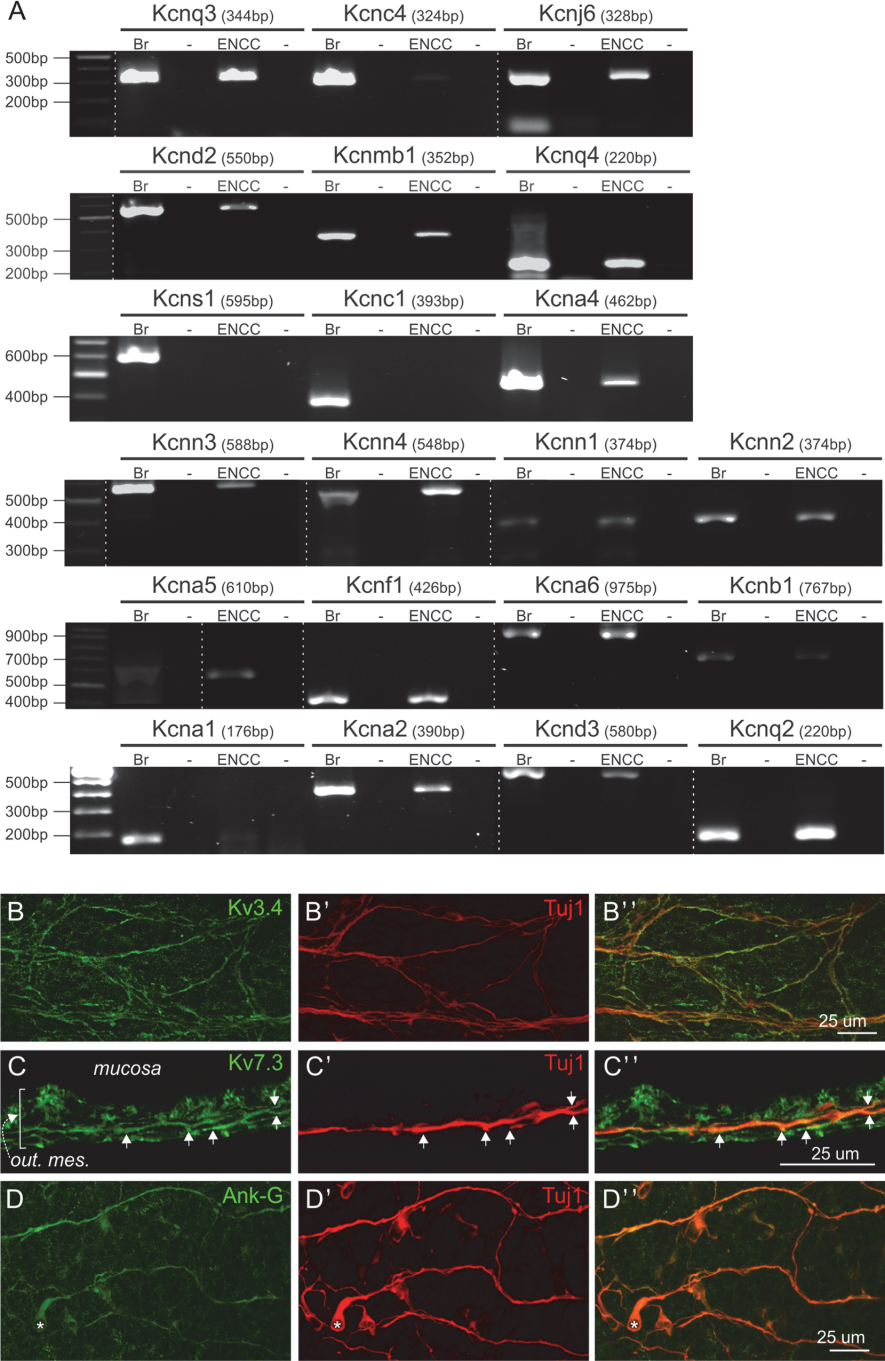
Expression of potassium channels and ankyrin-G by ENCCs. **A.** RT-PCR confirming expression of transcripts encoding 21 potassium channels in purified (FACS-sorted) ENCCs from E11.5 gut. Adult mouse brain (Br) was used as a positive control and-RT was a negative control (-). Dotted lines indicate different gels. **B.** Immunoreactivity for K_v_3.4 (**B**) (encoded by *Kcnc4*) in a wholemount preparation of E13.5 gut overlaps with Tuj1 immunostaining (**B’**, **B”**). **C.** Frozen transverse section of E14.5 gut: Immunoreactivity for K_v_7.3 (encoded by *Kcnq3*) overlaps with Tuj1 immunostaining of neurites (*arrows*, **C-C”**). Kv7.3 staining is also present in outer mesenchymal cells (*out mes*), but is not detectable in the mucosa (**C**). **D.** Immunolocalization of ankyrin-G in the E11.5 gut. Ankyrin-G immunostaining was present along the entire length of most Tuj1+ neurites (**D**-**D**”), and some nerve cell bodies were also ankyrin-G+ (*asterisk*).

Many potassium channels of various different subfamilies were expressed at E11.5 and most were upregulated significantly by E14.5, including *Kcnq3*, upregulated 170-fold, and *Kcnc4*, upregulated 16-fold (Tables 1 and 2). RT-PCR confirmed expression of these and other potassium channels at E14.5 (Fig 3A).

Immunohistochemistry was used to localize K_v_3.4 (*Kcnc4*), and K_v_7.3 (*Kcnq3*). Double staining of wholemount preparations of gut from E11.5, E12.5 and E13.5 mice with antibodies to K_v_3.4 and the neurite marker, Tuj1, showed that most Tuj1+ neurites close to the ENCC migratory wavefront showed K_v_3.4 immunoreactivity (Fig 3B-3B”). However, rostral to the wavefront, only a small sub-population of Tuj1+ neurites was K_v_3.4+. Only rarely was K_v_3.4 immunostaining detectable in Tuj1+ cell bodies. Frozen sections of embryonic gut immunostained using antibodies to K_v_7.3 revealed that Tuj1+ neurites showed K_v_7.3 staining (Fig 3C-3C”). Most cells in the outer mesenchymal layer were also K_v_7.3+, but were less intensely stained than the neurites (Fig 3C). Studies in the CNS have shown that K_v_7.2 and K_v_7.3 interact with ankyrin-G at the axon initial segment [54]. We therefore also examined the immunolocalization of ankyrin-G in the E11.5 and E12.5 gut. Double labelling with the neurite marker, Tuj1, showed that ankyrin-G immunostaining was present along the entire length of most neurites, and some nerve cell bodies were also ankyrin-G+ (Fig 3D-3D”).

We examined the effects of the non-selective K^+^ channel blockers, TEA and 4-AP [55], as well as the K_v_3.4 blocker, BDS-I, and the K_v_7 channel blocker, linopirdine, on ENCC migration and neurite outgrowth. The functional roles of K_v_.4.2, K_v_4.3, K_v_10.1 and K_v_11.1 were not examined in this study.

**Fig 4:**
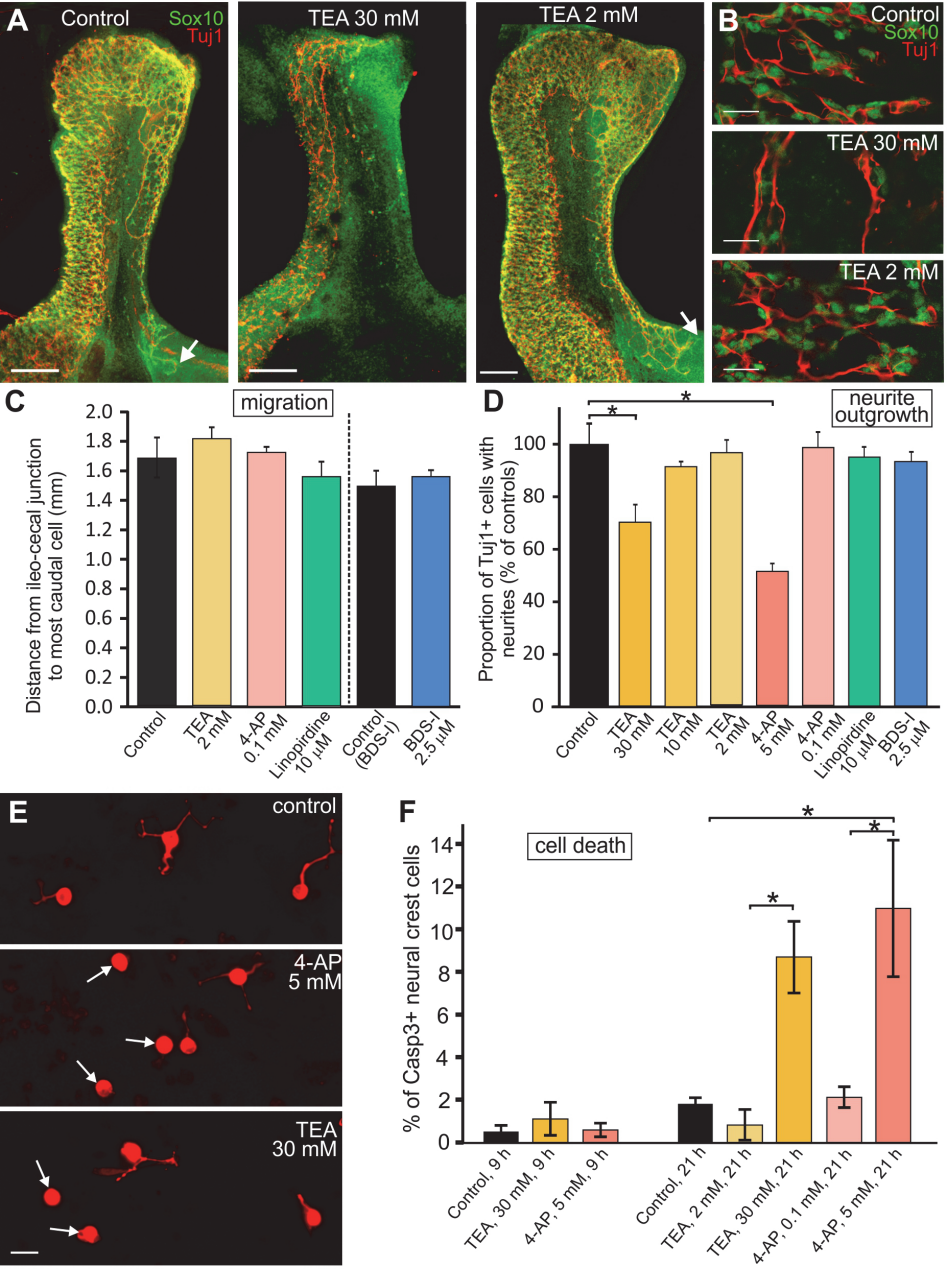
Effects of K^+^ channel blockers on ENCC migration and neuritogenesis. **A,B.** Explants of E11.5 gut were grown in control conditions or in the presence of K^+^ channel blockers for 48 h, and then fixed and processed for immunohistochemistry using antisera to the ENCC marker, Sox10 (green) and the neuronal marker, Tuj1 (red). **A.** In control explants and explants grown in the presence of 2 mM TEA, the most caudal Sox10+ ENCCs (*white arrows*) were close to the end of the explants. In explants grown in the presence of 30 mM TEA, there were very few Sox10+ ENCCs caudal to the caecum (middle panel). The red staining in the distal gut of the control explant is non-specific staining of the gut epithelial cells. Scale bars: 200 μm. **B.** High magnification images of the pre-caecal regions of E11.5 explants following 48 h in culture. There was a dramatic reduction in the number of Sox10+ cells (green) in gut explants grown in the presence of 30 mM TEA compared to control explants and explants grown in the presence of 2 mM TEA. Scale bars: 20 μm. **C.** Quantification of the location of the most caudal Sox10+ ENCC in E11.5 explants cultured for 48 h. Preparations exposed to 30 mM TEA or 5 mM 4-AP were not analysed due to the small number of Sox10+ cells in the post-caecal gut. There was no significant difference in the distance migrated by ENCCs in explants grown in the presence of 2 mM TEA (n = 8), 0.1 mM 4-AP (n = 10), the K_v_7 blocker, linopirdine (n = 11) or the K_v_3.4 blocker, BDS-I (n = 13) compared to controls (n = 12 or n = 14 for BDS-I experiments) (mean ± SEM; one way ANOVA; minimum of 2 experiments). The experiments using BDS-I were done separately from the other experiments and so had separate controls. Measurements were not performed on explants exposed to 30 mM TEA or 5 mM 4-AP due to the small number of surviving Sox10+ cells. **D.** Effects of potassium channel blockers on neurite outgrowth. The small and large intestines from E14.5 mice were dissociated, allowed to adhere to coverslips for 3 hours and then exposed to drugs for 9 h. The cells were then fixed and processed for immunohistochemistry using an antibody to Tuj1. A lower proportion of Tuj1+ cells extended neurites in the presence of 30 mM TEA or 5 mM 4-AP compared to controls (one way ANOVA followed by Tukeys test; for controls and each drug treatment, a minimum of 680 Tuj1+ cells was examined from 6 coverslips from 2 experiments). **E.** Examples of Tuj1+ cells in cultures of dissociated E14.5 gut grown under control conditions, or in the presence of 5 mM 4-AP or 30 mM TEA for 9 h. Under control conditions, most Tuj1+ cells extended neurites, whereas in the presence of 5 mM 4-AP or 30 mM TEA, many Tuj1+ cells did not possess any neurites (*arrows*). Scale bar: 20 μm. **F.** Gut from E14.5 *Ednrb-hKikGR* mice was dissociated, the cells allowed to adhere to coverslips for 3 hours and then exposed to drugs for 9 h or 21 h prior to fixation and processing for immunohistochemistry using the apoptosis marker, activated caspase-3. Although there was no difference in the proportions of KikGR+ ENCCs that were also activated caspase-3+ following 9 h exposure to 5 mM 4-AP or 30 mM TEA (one way ANOVA), there was a significant increase in activated caspase-3+ ENCCs after 21 h exposure to high concentrations of TEA or 4-AP (one way ANOVA followed by Tukeys test).

Previous studies showed that 4 mM 4-AP or 30 mM TEA inhibit the growth of retinal ganglion cells into the *Xenopus* brain [31]. Following exposure to 5 mM 4-AP or 30 mM TEA for 48 h, ENCCs in explants of E11.5 gut halted their caudal migration (Fig 4A), but there was also a striking decrease in the number of Sox10+ ENCCs (Fig 4B). In dissociated cultures of E14.5 gut grown in the presence of 30 mM TEA or 5 mM 4-AP for 9 h, the proportion of Tuj1+ cells extending neurites was significantly lower than controls (Fig 4D and 4E). Although there was no significant change in the proportion of activated caspase-3+ ENCCs in dissociated gut cultures after exposure to 30 mM TEA or 4 mM 4-AP for 9 h, there was a significant increase in the number of activated caspase-3+ ENCCs after 21 h exposure to the drugs (Fig 4F). The osmolarity of 30 mM TEA in TCM is ~16% higher than control TCM; however, the toxic effects of 30 mM TEA is not due to high osmolarity as the migration and survival of ENCCs in intact explants of E12.5 gut grown in TCM with 19% higher osmolality due to added sucrose did not differ from explants grown in control TCM (data not shown). Our data are consistent with very recent studies showing that exposure to high concentrations of TEA (>2 mM) kills HeLa cells [56] and cultured hippocampal neurons [57]. Lower concentrations of TEA (2 mM, 10 mM) or 4-AP (0.1 mM) had no significant effect on ENCC migration (Fig 4C), neuritogenesis (Fig 4D) or cell death (Fig 4F). Moreover, as in control gut explants, Tuj1+ neurites were closely associated with the most caudal Sox10+ cells. We could not quantify ENCC migration in explants exposed to 30 mM TEA or 5 mM 4-AP due to the small number of surviving Sox10+ cells (see Fig 4A).

*Kcnc4* (K_v_3.4) is upregulated ~16 fold between E11.5 and E14.5 (Table 1), and is expressed by newly extended neurites of young enteric neurons (see above). Although the K_v_3 subfamily of potassium channels is blocked by TEA and 4-AP [55], we also examined the effects of BDS-I, a specific blocker of K_v_3.4 [58]. BDS-I did not retard ENCC migration (Fig 4C) or neurite formation (Fig 4D).

*Kcnq3*, which encodes K_v_7.3, was upregulated 170 fold between E11.5 and E14.5 (Table 1). The K_v_7 subfamily of channels is not blocked by TEA or 4-AP; we therefore examined the effects of linopirdine, a blocker of K_v_7 channels [55], on ENCC migration and neurite extension. Linopirdine had no significant effect on ENCC migration or neuritogenesis (Fig 4C and 4D).

## Discussion

Using a PCR-based array and FACS-sorted ENCCs, we showed that a large number of ion channel genes are already expressed by ENCCs at E11.5, and expression of many ion channels increases significantly by E14.5. These ages span the time during which the colon is colonized by ENCCs and a subpopulation of ENCCs starts to differentiate into neurons and extends neurites [3,8,59,60].

The PCR array and RT-PCR studies were conducted on the entire population of ENCCs, including undifferentiated ENCCs and neurons. We therefore do not know whether particular ion channels are expressed by subpopulations of, or all, ENCCs. The up-regulation of numerous ion channels between E11.5 and E14.5 correlates with large increases in both the proportion of ENCCs expressing neuronal markers [7] and in enteric neuron density [9], so it is likely that many of the ion channels upregulated by E14.5 are expressed mainly or exclusively by neurons. This is supported by our immunohistochemical data showing that ion channels encoded by some of the most highly upregulated genes between E11.5 and E14.5 were localized predominantly to neurites; these include K_v_7.3 (*Kcnq3*, upregulated 170 fold), K_v_3.4 (*Kcnc4*, upregulated 16 fold) and Ca_v_2.2 (*Cacna1b*, upregulated 5 fold). However, although Ca_v_2.1 immunostaining was also predominantly on neurites, *Cacna1a* was expressed at similar levels at both E11.5 and E14.5. The ENS is one of the first parts of the nervous system to exhibit electrical activity [61]. We have previously shown that some ENCCs in the mouse gut express voltage-gated Ca^2+^ and Na^+^ channels that are involved in the electrical activity of early enteric neurons [35,39].

### Ion channels and ENCC migration

Ion channel activity is required for the migration of various types of cells, including glioma cells, platelets, neuroblasts and granule cells in the cerebellum [20,23–29]. Interestingly, there are some common mechanisms controlling the migration of CNS neural progenitors and the migration of glioma cells, including roles for several different ion channels [26]. It is thought that one mechanism by which ion channels facilitate cell migration is by promoting hydrodynamic volume and shape changes that enable cells to squeeze between small spaces [20,62]. Since ENCCs undergo rapid changes in shape as they migrate [13,63–65], we hypothesized that ion channel activity might be required for ENCC migration. We found that high concentrations of the non-selective K+ channel inhibitors, TEA and 4-AP, halted ENCC migration in cultured explants, but killed the vast majority of ENCCs. In contrast, addition of drugs that block the activities of a range of Ca^2+^, Cl^-^ and K^+^ channels at concentrations that did not cause cell death did not perturb the migration of ENCCs in gut explants. The lack of effect of the channel-blocking drugs on ENCC migration is unlikely to be due to poor penetration of the drugs as intact E11.5 gut explants are permeable to antibodies and large (250 kDa) toxins [47,66], and often similar concentrations of drugs are used to affect ENCCs in intact embryonic gut explants to those used in dissociated ENCCs [64,67]. We had previously shown that tetrodotoxin, which blocks voltage-gated sodium channels, and clotimazole, which blocks the intermediate conductance Ca^2+^-dependent K^+^ channel (K_Ca_3.1), also do not affect ENCC migration [50]. It is possible that it is necessary to block multiple ion channels simultaneously to perturb ENCC migration and/or other types of ion channels not tested are involved in ENCC migration. For example, Kv11.1 is involved in the migration of some non-neuronal cell types [68], but its function was not examined in the current study. It is also feasible that ENCCs do not require ion channel activity but use other mechanisms to migrate through the gut mesenchyme. For example, blocking matrix metalloproteinase activity has been shown to retard ENCC migration [69].

### Ion channels and neurite extension

At the same time as ENCCs are migrating along the gut, a sub-population of cells starts to differentiate into neurons and extends neurites [10,12,59]. During formation of the ENS, it is essential that developing neurons project their axons in the correct direction and to the correct targets. Little is known about the mechanisms controlling axon extension and navigation in the developing ENS, although the planar cell polarity (PCP) pathway has been shown to play a role in the direction in which axons project along the gut *in vivo* [12], and retinoic acid retards neurite extension from enteric neurons *in vitro* [70]. Ion channels, including Ca^2+^ channels [32,34], Cl^-^ channels [33] and K^+^ channels [71], have been reported to regulate axon outgrowth in a variety of regions of the nervous system.

Many voltage-dependent K^+^ channels are expressed in the developing *Xenopus* brain [71], and K_v_3.4 is expressed by pioneer axons in the developing rat forebrain [72]. Similarly, we showed that K_v_3.4 immunoreactivity in the developing gut was most prominent on neurites close to the migratory wavefront.

Exposure of dissociated ENCCs to high concentrations of the non-selective K^+^ channel blockers, TEA (30 mM) and 4-AP (5mM) for 9 hours resulted in a smaller percentage of Tuj1+ cells extending neurites *in vitro*. However, although similar concentrations of these drugs have been used previously in studies of axon growth and guidance in the *Xenopus* visual system, we found that TEA and 4-AP at high concentrations caused death of ENCCs; no increase in cell death (activated caspase-3+ cells) was detectable after only 9 hours exposure to 30 mM TEA or 5 mM 4-AP, but exposure to the drugs for 21 hours did result in a significant increase in cell death. Furthermore, there was a dramatic reduction in the number of ENCCs in intact explants of E11.5 gut following exposure to 30 mM TEA or 5 mM 4-AP for 48 h for the ENCC migration assays (see above). It is therefore highly likely that the decreased neuritogenesis observed after 9 h exposure to 30 mM TEA or 5 mM 4-AP is due to ENCCs being unhealthy. Other recent studies using HeLa cells and cultured hippocampal neurons have also reported that exposure to high concentrations of TEA for 24 h or more results in cell death [56,57]. Proteomics analyses of HeLa cells exposed to high concentrations of TEA revealed changes in proteins involved in a variety of biological functions including oxidative stress responses, protein synthesis and degradation, metabolism and signal transduction [56].

Decreased neuritogenesis was observed after 9 hours exposure to high concentrations of TEA or 4-AP, but no significant cell death was detected at this time point. Although we think the decreased neuritogenesis observed after 9 hours is most likely due to the cells being unhealthy, we cannot rule out the possibility that the perturbed neuritogenesis is a primary effect of exposure to high concentrations of TEA or 4-AP, with prolonged drug exposure then leading to extra effects and ultimately cell death.

Different neurochemical classes of neurons are present in the E14.5 gut [9,50]. Although there was an overall reduction in the proportion of Tuj1+ cells extending neurites after 9 hours exposure to high concentrations of TEA or 4-AP, some neurons still extended neurites (see Fig 4E). As Tuj1 is a pan-neuronal marker, it is possible that different classes of neurons are differentially sensitive to TEA and 4-AP.

Despite numerous studies showing roles for ion channel activity in neurite extension in a variety of regions of the nervous system, we were unable to demonstrate a role for ion channel activity in neuritogenesis in cultured embryonic enteric neurons. We also did not observe any obvious changes to the projections of Tuj1+ neurites in intact explants of gut in the ENCC migration assays. We cannot rule out the possibilities that other ion channels are involved, that it is necessary to block multiple ion channels simultaneously to perturb neurite formation or that ion channels play a role in enteric neuritogenesis (both axon and dendrites) *in vivo* but not *in vitro*. The axons of enteric neurons have to navigate to specific targets, including the external muscle and other ganglia and it is possible ion channels are involved in axon navigation or synaptogenesis *in vivo*. Furthermore, enteric neurons undergo significant morphological changes in their dendrites during development [41] but the mechanisms underlying these dendritic changes are unknown.

### Ion channels and electrical excitability

We have previously shown functional roles for several Na^+^, Ca^2+^ and K^+^ channels in the development of electrical excitability in early enteric neurons [35,39], and it is possible that the vast majority of ion channels expressed in the fetal ENS are involved in the excitability of all, or specific subtypes of, enteric neurons [60].

In the CNS, ankyrin-G is concentrated at axon initial segments and plays an essential role in the clustering and function of K_v_7.2 and K_v_7.3 [54]. In the current study, we showed that ankyrin-G and K_v_7.3 were expressed by many embryonic enteric neurons, but appeared to be present along the entire length of the neurites. Na_v_1.6 has been reported to localize to the axon initial segments of enteric neurons in the colon of adult guinea-pigs [73], but little else is known about the axon initial segments in the ENS. Even though embryonic enteric neurons can fire action potentials, our ankyrin-G data suggest that they may not yet possess axon initial segments.

## Conclusions

Our array data showed that many ion channels are expressed early during the development of the ENS. Many ion channels are likely to be involved in the development of neuronal electrical excitability, but it remains to be determined which ion channels are expressed by undifferentiated ENCCs and whether they also play functional roles.

## Supporting Information

S1 Table Primer sequences and the TD-PCR cycling program used for ion channel amplification. (DOCX)

S2 Table TD-PCR cycling program parameters. Adapted from [74].
(DOCX)

S3 Table Primary antisera. (DOCX)

S4 Table Secondary antisera. (DOCX)

S5 Table Expression of ion channels at E11.5, E14.5, P0 and Adult. (XLS)

## References

[1] Yntema CL, Hammond WS (1954) The origin of intrinsic ganglia of trunk viscera from vagal neural crest in the chick embryo. J Comp Neurol 101: 515–541. 13221667 10.1002/cne.901010212

[2] Burns AJ, Le Douarin NM (1998) The sacral neural crest contributes neurons and glia to the post- umbilical gut: spatiotemporal analysis of the development of the enteric nervous system. Development 125: 4335–4347. 9753687 10.1242/dev.125.21.4335

[3] Kapur RP, Yost C, Palmiter RD (1992) A transgenic model for studying development of the enteric nervous system in normal and aganglionic mice. Development 116: 167–175. 1483385 10.1242/dev.116.Supplement.167

[4] Young HM, Hearn CJ, Ciampoli D, Southwell BR, Brunet JF, Newgreen DF (1998) A single rostrocaudal colonization of the rodent intestine by enteric neuron precursors is revealed by the expression of Phox2b, Ret, and p75 and by explants grown under the kidney capsule or in organ culture. Dev Biol 202: 67–84. 9758704 10.1006/dbio.1998.8987

[5] Obermayr F, Hotta R, Enomoto H, Young HM (2013) Development and developmental disorders of the enteric nervous system. Nat Rev Gastroenterol Hepatol 10: 43–57. 10.1038/nrgastro.2012.234 23229326

[6] Baetge G, Schneider KA, Gershon MD (1990) Development and persistence of catecholaminergic neurons in cultured explants of fetal murine vagus nerves and bowel. Development 110: 689–701. 1982430 10.1242/dev.110.3.689

[7] Young HM, Bergner AJ, Muller T (2003) Acquisition of neuronal and glial markers by neural crest-derived cells in the mouse intestine. J Comp Neurol 456: 1–11. 12508309 10.1002/cne.10448

[8] Hao MM, Anderson RB, Kobayashi K, Whitington PM, Young HM (2009) The migratory behavior of immature enteric neurons. Dev Neurobiol 69: 22–35. 10.1002/dneu.20683 18985707

[9] Erickson CS, Lee SJ, Barlow-Anacker AJ, Druckenbrod NR, Epstein ML, Gosain A (2014) Appearance of cholinergic myenteric neurons during enteric nervous system development: comparison of different ChAT fluorescent mouse reporter lines. Neurogastroenterol Motil 26: 874–884. 10.1111/nmo.12343 24712519 PMC4037379

[10] Young HM, Jones BR, McKeown SJ (2002) The projections of early enteric neurons are influenced by the direction of neural crest cell migration. J Neurosci 22: 6005–6018. 12122062 10.1523/JNEUROSCI.22-14-06005.2002 PMC6757928

[11] Nishiyama C, Uesaka T, Manabe T, Yonekura Y, Nagasawa T, Newgreen DF, et al (2012) Trans-mesenteric neural crest cells are the principal source of the colonic enteric nervous system. Nat Neurosci 15: 1211–1218. 10.1038/nn.3184 22902718

[12] Sasselli V, Boesmans W, Vanden Berghe P, Tissir F, Goffinet AM, Pachnis V (2013) Planar cell polarity genes control the connectivity of enteric neurons. J Clin Invest 123: 1763–1772. 10.1172/JCI66759 23478408 PMC3613929

[13] Young HM, Bergner AJ, Simpson MJ, McKeown SJ, Hao MM, Anderson CR, et al (2014) Colonizing while migrating: how do individual enteric neural crest cells behave? BMC Biol 12: 23 10.1186/1741-7007-12-23 24670214 PMC4101823

[14] Gershon MD (2010) Developmental determinants of the independence and complexity of the enteric nervous system. Trends Neurosci 33: 446–456. 10.1016/j.tins.2010.06.002 20633936

[15] Laranjeira C, Pachnis V (2009) Enteric nervous system development: Recent progress and future challenges. Auton Neurosci 151: 61–69. 10.1016/j.autneu.2009.09.001 19783483

[16] Lake JI, Heuckeroth RO (2013) Enteric Nervous System Development: Migration, Differentiation, and Disease. Am J Physiol Gastrointest Liver Physiol 305: G1–24. 10.1152/ajpgi.00452.2012 23639815 PMC3725693

[17] Pardo LA, Stuhmer W (2014) The roles of K(+) channels in cancer. Nat Rev Cancer 14: 39–48. 10.1038/nrc3635 24336491

[18] Yu PC, Du JL (2011) Transient receptor potential canonical channels in angiogenesis and axon guidance. Cell Mol Life Sci 68: 3815–3821. 10.1007/s00018-011-0755-x 21755360 PMC11114694

[19] Stock C, Ludwig FT, Hanley PJ, Schwab A (2013) Roles of ion transport in control of cell motility. Compr Physiol 3: 59–119. 10.1002/cphy.c110056 23720281

[20] Cuddapah VA, Sontheimer H (2011) Ion channels and transporters [corrected] in cancer. 2. Ion channels and the control of cancer cell migration. Am J Physiol Cell Physiol 301: C541–549. 10.1152/ajpcell.00102.2011 21543740 PMC3174565

[21] Schwab A, Hanley P, Fabian A, Stock C (2008) Potassium channels keep mobile cells on the go. Physiology (Bethesda) 23: 212–220. 10.1152/physiol.00003.2008 18697995

[22] Becchetti A, Arcangeli A (2010) Integrins and ion channels in cell migration: implications for neuronal development, wound healing and metastatic spread. Adv Exp Med Biol 674: 107–123. 20549944 10.1007/978-1-4419-6066-5_10

[23] Lui VC, Lung SS, Pu JK, Hung KN, Leung GK (2010) Invasion of human glioma cells is regulated by multiple chloride channels including ClC-3. Anticancer Res 30: 4515–4524. 21115901

[24] Cuddapah VA, Turner KL, Seifert S, Sontheimer H (2013) Bradykinin-induced chemotaxis of human gliomas requires the activation of KCa3.1 and ClC-3. J Neurosci 33: 1427–1440. 10.1523/JNEUROSCI.3980-12.2013 23345219 PMC3711544

[25] D'Alessandro G, Catalano M, Sciaccaluga M, Chece G, Cipriani R, Rosito M, et al (2013) KCa3.1 channels are involved in the infiltrative behavior of glioblastoma in vivo. Cell Death Dis 4: e773 10.1038/cddis.2013.279 23949222 PMC3763441

[26] Cuddapah VA, Robel S, Watkins S, Sontheimer H (2014) A neurocentric perspective on glioma invasion. Nat Rev Neurosci 15: 455–465. 10.1038/nrn3765 24946761 PMC5304245

[27] Schmidt EM, Munzer P, Borst O, Kraemer BF, Schmid E, Urban B, et al (2011) Ion channels in the regulation of platelet migration. Biochem Biophys Res Commun 415: 54–60. 10.1016/j.bbrc.2011.10.009 22005466

[28] Turner KL, Sontheimer H (2013) KCa3.1 Modulates Neuroblast Migration Along the Rostral Migratory Stream (RMS) In Vivo. Cereb Cortex. 10.1093/cercor/bht090 PMC4128704 23585521

[29] Komuro H, Rakic P (1992) Selective role of N-type calcium channels in neuronal migration. Science 257: 806–809. 1323145 10.1126/science.1323145

[30] Moran D (1991) Voltage-dependent-L-type Ca2+ channels participate in regulating neural crest migration and differentiation. Am J Anat 192: 14–22. 1661064 10.1002/aja.1001920103

[31] McFarlane S, Pollock NS (2000) A role for voltage-gated potassium channels in the outgrowth of retinal axons in the developing visual system. J Neurosci 20: 1020–1029. 10648707 10.1523/JNEUROSCI.20-03-01020.2000 PMC6774185

[32] Mire E, Mezzera C, Leyva-Diaz E, Paternain AV, Squarzoni P, Bluy L, et al (2012) Spontaneous activity regulates Robo1 transcription to mediate a switch in thalamocortical axon growth. Nat Neurosci 15: 1134–1143. 10.1038/nn.3160 22772332

[33] Hur J, Jeong HJ, Park J, Jeon S (2013) Chloride channel 4 is required for nerve growth factor-induced TrkA signaling and neurite outgrowth in PC12 cells and cortical neurons. Neuroscience 253: 389–397. 10.1016/j.neuroscience.2013.09.003 24036377

[34] Sann SB, Xu L, Nishimune H, Sanes JR, Spitzer NC (2008) Neurite outgrowth and in vivo sensory innervation mediated by a Ca(V)2.2-laminin beta 2 stop signal. J Neurosci 28: 2366–2374. 10.1523/JNEUROSCI.3828-07.2008 18322083 PMC6671202

[35] Hao MM, Lomax AE, McKeown SJ, Reid CA, Young HM, Bornstein JC (2012) Early development of electrical excitability in the mouse enteric nervous system. J Neurosci 32: 10949–10960. 10.1523/JNEUROSCI.1426-12.2012 22875929 PMC6621017

[36] Heanue TA, Pachnis V (2006) Expression profiling the developing mammalian enteric nervous system identifies marker and candidate Hirschsprung disease genes. Proc Natl Acad Sci U S A 103: 6919–6924. 16632597 10.1073/pnas.0602152103 PMC1458994

[37] Vohra BP, Tsuji K, Nagashimada M, Uesaka T, Wind D, Fu M, et al (2006) Differential gene expression and functional analysis implicate novel mechanisms in enteric nervous system precursor migration and neuritogenesis. Dev Biol 298: 259–271. 16904662 10.1016/j.ydbio.2006.06.033 PMC1952185

[38] Hotta R, Stamp LA, Foong JP, Bergner AJ, McConnell SN, Anderson RB, et al (2013) Transplanted progenitors generate functional enteric neurons in the postnatal colon J Clin Invest 123: 1182–1191. 10.1172/JCI65963 23454768 PMC3582137

[39] Hao MM, Boesmans W, Van den Abbeel V, Jennings EA, Bornstein JC, Young HM, et al (2011) Early emergence of neural activity in the developing mouse enteric nervous system. J Neurosci 31: 15352–15361. 10.1523/JNEUROSCI.3053-11.2011 22031881 PMC6703522

[40] Park KJ, Hennig GW, Lee HT, Spencer NJ, Ward SM, Smith TK, et al (2006) Spatial and temporal mapping of pacemaker activity in interstitial cells of Cajal in mouse ileum in situ. Am J Physiol Cell Physiol 290: C1411–1427. 16381798 10.1152/ajpcell.00447.2005

[41] Foong JP, Nguyen TV, Furness JB, Bornstein JC, Young HM (2012) Myenteric neurons of the mouse small intestine undergo significant electrophysiological and morphological changes during postnatal development. J Physiol 590: 2375–2390. 10.1113/jphysiol.2011.225938 22371477 PMC3424759

[42] Ubels JL, Schotanus MP, Bardolph SL, Haarsma LD, Koetje LR, Louters JR (2010) Inhibition of UV-B induced apoptosis in corneal epithelial cells by potassium channel modulators. Exp Eye Res 90: 216–222. 10.1016/j.exer.2009.10.005 19874821 PMC2822080

[43] Sun J, Kapur J (2012) M-type potassium channels modulate Schaffer collateral-CA1 glutamatergic synaptic transmission. J Physiol 590: 3953–3964. 10.1113/jphysiol.2012.235820 22674722 PMC3476642

[44] Hearn CJ, Young HM, Ciampoli D, Lomax AE, Newgreen D (1999) Catenary cultures of embryonic gastrointestinal tract support organ morphogenesis, motility, neural crest cell migration, and cell differentiation. Dev Dyn 214: 239–247. 10090150 10.1002/(SICI)1097-0177(199903)214:3<239::AID-AJA7>3.0.CO;2-O

[45] Southard-Smith EM, Kos L, Pavan WJ (1998) Sox10 mutation disrupts neural crest development in Dom Hirschsprung mouse model. Nat Genet 18: 60–64. 9425902 10.1038/ng0198-60

[46] Barlow AJ, Wallace AS, Thapar N, Burns AJ (2008) Critical numbers of neural crest cells are required in the pathways from the neural tube to the foregut to ensure complete enteric nervous system formation. Development 135: 1681–1691. 10.1242/dev.017418 18385256

[47] Anderson RB, Turner KN, Nikonenko AG, Hemperly J, Schachner M, Young HM (2006) The cell adhesion molecule L1 is required for chain migration of neural crest cells in the developing mouse gut. Gastroenterology 130: 1221–1232. 16618414 10.1053/j.gastro.2006.01.002

[48] Bergner AJ, Stamp LA, Gonsalvez DG, Allison MB, Olson DP, Myers MG, et al (2014) Birthdating of myenteric neuron subtypes in the small intestine of the mouse. J Comp Neurol 522: 514–527. 10.1002/cne.23423 23861145 PMC3877185

[49] Pan Z, Kao T, Horvath Z, Lemos J, Sul JY, Cranstoun SD, et al (2006) A common ankyrin-G-based mechanism retains KCNQ and NaV channels at electrically active domains of the axon. J Neurosci 26: 2599–2613. 16525039 10.1523/JNEUROSCI.4314-05.2006 PMC6675151

[50] Hao MM, Moore RE, Roberts RR, Nguyen T, Furness JB, Anderson RB, et al (2010) The role of neural activity in the migration and differentiation of enteric neuron precursors. Neurogastroenterol Motil 22: e127–137. 10.1111/j.1365-2982.2009.01462.x 20082666

[51] McFerrin MB, Sontheimer H (2006) A role for ion channels in glioma cell invasion. Neuron Glia Biol 2: 39–49. 16520829 10.1017/S17440925X06000044 PMC1389710

[52] Habela CW, Ernest NJ, Swindall AF, Sontheimer H (2009) Chloride accumulation drives volume dynamics underlying cell proliferation and migration. J Neurophysiol 101: 750–757. 10.1152/jn.90840.2008 19036868 PMC2657062

[53] Hilaire C, Diochot S, Desmadryl G, Baldy-Moulinier M, Richard S, Valmier J (1996) Opposite developmental regulation of P- and Q-type calcium currents during ontogenesis of large diameter mouse sensory neurons. Neuroscience 75: 1219–1229. 8938755 10.1016/0306-4522(96)00347-8

[54] Cooper EC (2011) Made for "anchorin": Kv7.2/7.3 (KCNQ2/KCNQ3) channels and the modulation of neuronal excitability in vertebrate axons. Semin Cell Dev Biol 22: 185–192. 10.1016/j.semcdb.2010.10.001 20940059 PMC3070838

[55] Gu C, Barry J (2011) Function and mechanism of axonal targeting of voltage-sensitive potassium channels. Prog Neurobiol 94: 115–132. 10.1016/j.pneurobio.2011.04.009 21530607 PMC3112463

[56] Huang L, Huang QY, Huang HQ (2014) The evidence of HeLa cell apoptosis induced with tetraethylammonium using proteomics and various analytical methods. J Biol Chem 289: 2217–2229. 10.1074/jbc.M113.515932 24297172 PMC3900967

[57] Chen M, Sun HY, Hu P, Wang CF, Li BX, Li SJ, et al (2013) Activation of BKca channels mediates hippocampal neuronal death after reoxygenation and reperfusion. Mol Neurobiol 48: 794–807. 10.1007/s12035-013-8467-x 23653329

[58] Diochot S, Schweitz H, Beress L, Lazdunski M (1998) Sea anemone peptides with a specific blocking activity against the fast inactivating potassium channel Kv3.4. J Biol Chem 273: 6744–6749. 9506974 10.1074/jbc.273.12.6744

[59] Baetge G, Gershon MD (1989) Transient catecholaminergic (TC) cells in the vagus nerves and bowel of fetal mice: relationship to the development of enteric neurons. Dev Biol 132: 189–211. 2563710 10.1016/0012-1606(89)90217-0

[60] Hao MM, Young HM (2009) Development of enteric neuron diversity. J Cell Mol Med 13: 1193–1210. 10.1111/j.1582-4934.2009.00813.x 19538470 PMC4496134

[61] Hao MM, Bornstein JC, Vanden Berghe P, Lomax AE, Young HM, Foong JP (2013) The emergence of neural activity and its role in the development of the enteric nervous system. Dev Biol 382: 365–374. 10.1016/j.ydbio.2012.12.006 23261929

[62] Cuddapah VA, Turner KL, Sontheimer H (2013) Calcium entry via TRPC1 channels activates chloride currents in human glioma cells. Cell Calcium 53: 187–194. 10.1016/j.ceca.2012.11.013 23261316 PMC3594368

[63] Druckenbrod NR, Epstein ML (2007) Behavior of enteric neural crest-derived cells varies with respect to the migratory wavefront. Dev Dyn 236: 84–92. 17039523 10.1002/dvdy.20974

[64] Druckenbrod NR, Epstein ML (2009) Age-dependent changes in the gut environment restrict the invasion of the hindgut by enteric neural progenitors. Development 136: 3195–3203. 10.1242/dev.031302 19700623

[65] Xu Q, Heanue T, Pachnis V (2014) Travelling within the fetal gut: simple rules for an arduous journey. BMC Biol 12: 50 10.1186/s12915-014-0050-z 25184534 PMC4096386

[66] Stewart AL, Young HM, Popoff M, Anderson RB (2007) Effects of pharmacological inhibition of small GTPases on axon extension and migration of enteric neural crest-derived cells. Dev Biol 307: 92–104. 17524389 10.1016/j.ydbio.2007.04.024

[67] Gisser JM, Cohen AR, Yin H, Gariepy CE (2013) A novel bidirectional interaction between endothelin-3 and retinoic acid in rat enteric nervous system precursors. PLoS One 8: e74311 10.1371/journal.pone.0074311 24040226 PMC3767828

[68] Wulff H, Castle NA, Pardo LA (2009) Voltage-gated potassium channels as therapeutic targets. Nat Rev Drug Discov 8: 982–1001. 10.1038/nrd2983 19949402 PMC2790170

[69] Anderson RB (2010) Matrix metalloproteinase-2 is involved in the migration and network formation of enteric neural crest-derived cells. Int J Dev Biol 54: 63–69. 10.1387/ijdb.082667ra 19247964

[70] Sato Y, Heuckeroth RO (2008) Retinoic acid regulates murine enteric nervous system precursor proliferation, enhances neuronal precursor differentiation, and reduces neurite growth in vitro. Dev Biol 320: 185–198. 10.1016/j.ydbio.2008.05.524 18561907 PMC2586054

[71] Pollock NS, Ferguson SC, McFarlane S (2002) Expression of voltage-dependent potassium channels in the developing visual system of Xenopus laevis. J Comp Neurol 452: 381–391. 12355420 10.1002/cne.10401

[72] Huang CY, Chu D, Hwang WC, Tsaur ML (2012) Coexpression of high-voltage-activated ion channels Kv3.4 and Cav1.2 in pioneer axons during pathfinding in the developing rat forebrain. J Comp Neurol 520: 3650–3672. 10.1002/cne.23119 22473424

[73] Bartoo AC, Sprunger LK, Schneider DA (2005) Expression and distribution of TTX-sensitive sodium channel alpha subunits in the enteric nervous system. J Comp Neurol 486: 117–131. 15844213 10.1002/cne.20541

[74] Korbie DJ, Mattick JS (2008) Touchdown PCR for increased specificity and sensitivity in PCR amplification. Nat Protoc 3: 1452–1456. 10.1038/nprot.2008.133 18772872

