# Supplementary material for: Ion Channel Expression in the Developing Enteric Nervous System
Source: PLoS One. 2015 Mar 23;10(3):e0123436. doi: 10.1371/journal.pone.0123436 (PMC4370736; doi:10.1371/journal.pone.0123436)
Supplement: S1 Table — (DOCX) [file pone.0123436.s001.docx]

**S1 Table.** **Primer sequences and the TD-PCR cycling program used for ion channel amplification.**

| **Gene Symbol** | **Forward Primer Sequence (5’🡪3’)** | **Reverse Primer Sequence (5’🡪3’)** | **TD PCR Program**  **Brain ENCS** | |
| --- | --- | --- | --- | --- |
| Cacna1a | TCT CTG GGC CGA TAC ACT GA | CGG AAC TAC TGC CCT GTC TG | TDa | TDb |
| Cacna1b | CTG GTG GCA TTT GCG TTC TC | CCA AAT ACT GAC CCC GGC A | TDa | TDb |
| Cacna1c | TTC GCT CTG CCT CTC TAG GT | ATC AAG ACC GCT TCC ACC AG | TDa | TDa |
| Cacna1h | AGC ACT ACA ACC AGC CCA AG | TCC TCG CTG CAT TCT AGC CT | TDa | TDa |
| Cacnb1 | GCT CAA GGG CTA TGA GGT GA | GCT TCT CCG AGG CTG CTA TT | TDa | TDa |
| Cacnb3 | GCT GGA AAG AGC CAA GCA CA | CAT GGA GGG CAC CAC ATC AT | TDa | TDa |
| Cacnb4 | CCC AAA GGT TTT ACA GCG GC | CAT AGG GGT GCT ACT GCT CG | TDa | TDb |
| Cacng2 | CTG CCT CGA AGG GAA CTT CA | GGC GGT CGA TAA ACA TGT GC | TDa | TDb |
| Clcn2 | AGA TTG TCC AGG TGA TGC GG | TGC CGC TCC AAT GAC AAA GA | TDa | TDa |
| Clcn3 | TGC AAT AGA AAG TGC CAG AA | ACT CTT TCC CTT CTC CCT CC | TDa | TDa |
| Clcn4-2 | GGC ATT TAG AAG CAC CAC GC | TGC TGG TGA TCT TCC TAT GTC TG | TDa | TDb |
| Clcn5 | ATG TAC GTC CTC TGG GCT CT | TAG CTG ACC TCT TCC AGG CT | TDa | TDa |
| Clcn6 | GGA AAG TCA GTC CAG TGC CA | GTGCCG TGTAAT GAT CCCCA | TDa | TDa |
| Clcn7 | CTC AGC TTG GTG GGA TCG TG | AAC GAT AAG CTG GGA ACG CA | TDa | TDa |
| Kcna1 | CTC GTT CCG GCT GCT GCA AA | TCA CCC ACT TTG CTG CTC CTT | TDa | TDa |
| Kcna2 | GCT CTG GTA CCC ATC TGC AA | CCC AGA GCC CTT TGT GAG TT | TDa | TDa |
| Kcna4 | ACC AGA AAT CTC CGG GGT CT | CAA GCC TTC ATG GAC CCA CA | TDa | TDa |
| Kcna5 | AGG AGG TTT TTC TGC TGC CTG | TCA AAG CGC AAA CCC GAG AT | TDb | TDa |
| Kcna6 | TCA TCA CTG TCC CTG TCC TTA TG | GGT TTT CCA GAG TGG CCT CG | TDa | TDa |
| Kcnb1 | TGG AGA AGC CCA ACT CAT CG | CTC TCT CCG CTT GAT GGC TT | TDa | TDa |
| Kcnc1 | GGA TGA CCT ATC GCC AGC AC | GGT AGT ACC GCA CTT GGG TG | TDa | TDa |
| Kcnc4 | CCC ATT TAC TGC AAG TCT GAG GA | CGG CTC AAC ATT GCC TTC TTT C | TDa | TDa |
| Kcnd2 | TGA CAA CAC TGG GGT ATG GC | CCT CTA TGG CTT CCC GAC AC | TDb | TDb |
| Kcnd3 | AGA TAC CGC TTC ATC CGC AG | TGT GCA GGT AGG CAT TGG AG | TDb | TDb |
| Kcnf1 | TAG GAG CCG TAG AGC CTT GT | TCC CCT AGG ATG GCC ATT GA | TDb | TDb |
| Kcnj6 | GTT CCG AGA GAG GCG ATC AG | CCA GGG TGG TGA AGA TGT CC | TDa | TDa |
| Kcnmb1 | AGT ACC CAT GCC TTT GGG TC | TGA GCC GCC AAG ATG GAT AG | TDb | TDb |
| Kcnn1 | AAC TTC CTG GGA GCC ATG TG | ACA CTT CGG AGC TTC TGA GC | TDa | TDa |
| Kcnn2 | CGA CAA GGC GTC GCT GTA TT | ACG AGG CAT CGG TGA AAA GT | TDa | TDa |
| Kcnn3 | CCC CAT CCC TGG AGA GTA CA | GCG GCA TTC TTG ATC CGT TT | TDa | TDa |
| Kcnn4 | TGA CCA TTG GCT ATG GGG AC | CAT GTG GAG CTA TGT GGC CT | TDb | TDb |
| Kcnq2 | CCT GGA AGC TCT TGG GAT CG | CCG TTC CAG GTC TGA GGG TA | TDb | TDb |
| Kcnq3 | CCA GCA GTC TCC AAG GAA TGA | AAG GGC CAA CTC TGT CGA TG | TDa | TDa |
| Kcnq4 | ATC TTT CAG AGA GCT GGC CC | GCG GAT TCG GTC TTT GAT GC | TDb | TDb |
| Kcns1 | ATT GAC ATC GTG TCG GTG CT | AAG TGT CCC GGG ATG TTT CC | TDa | TDa |
| Cacna1a | TCT CTG GGC CGA TAC ACT GA | CGG AAC TAC TGC CCT GTC TG | TDa | TDb |
