# Supplementary material for: Ion Channel Expression in the Developing Enteric Nervous System
Source: PLoS One. 2015 Mar 23;10(3):e0123436. doi: 10.1371/journal.pone.0123436 (PMC4370736; doi:10.1371/journal.pone.0123436)
Supplement: S2 Table — Adapted from [74]. (DOCX) [file pone.0123436.s002.docx]

**S2 Table.** **TD-PCR cycling program parameters**. Adapted from [[74](#_ENREF_74)].

| **Phase 1 Step Temperature Time** | | | |
| --- | --- | --- | --- |
| 1 | Denature | 95^◦^C | 5 min |
| 2 | Denature | 95^◦^C | 30 s |
| 3 | Anneal | 65^◦^C | 45 s |
| 4 | Elongate | 72^◦^C | 30 s |
| Repeat 2-4 (9 times) reducing annealing temperature by 1^◦^C/cycle | | | |
| **Phase 2 Step Temperature Time** | | | |
| **5** | Denature | 95^◦^C | 30 s |
| 6 | Anneal | 55^◦^C | 45 s |
| 7 | Elongate | 72^◦^C | 30 s |
| Repeat steps 5-6 either 24 times (TDa) or 29 times (TDb) | | | |
| **Termination Step Temperature Time** | | | |
| 8 | Elongate | 72^◦^C | 10 min |
| 9 | Halt reaction | 4^◦^C | 10 min |
