# Supplementary material for: Ion Channel Expression in the Developing Enteric Nervous System
Source: PLoS One. 2015 Mar 23;10(3):e0123436. doi: 10.1371/journal.pone.0123436 (PMC4370736; doi:10.1371/journal.pone.0123436)
Supplement: S3 Table — (DOCX) [file pone.0123436.s003.docx]

**S3 Table. Primary antisera**

| Primary Antibodies Used | | | |
| --- | --- | --- | --- |
| **Antigen** | **Host Species** | **Dilution** | **Supplier/Reference** |
| Sox10 | Goat | 1:100 | Santa Cruz Biotechnology, Santa Cruz, CA, USA |
| β-tubulin 3 (Tuj1) | Mouse | 1:2000 | Covance, Princeton, NJ, USA |
| α_1A_ (Ca_v_2.1)  α_1B_ (Ca_v_2.2)  KCNC4 (K_v_3.4)  KCNQ3N (K_v_7.3)  Ankyrin-G | Rabbit  Rabbit  Rabbit  Guinea Pig  Rabbit | 1:50  1:50  1:50  1:100  1:500 | Alomone Labs, Jerusalem, Israel  Alomone Labs  Alomone Labs  [[49](#_ENREF_49)]  Santa Cruz |
| Activated caspase-3  Tuj1 | Rabbit  Mouse | 1:1000  1:100 | R&D Systems, Minneapolis, MN, USA  Covance |
