# Supplementary material for: Ion Channel Expression in the Developing Enteric Nervous System
Source: PLoS One. 2015 Mar 23;10(3):e0123436. doi: 10.1371/journal.pone.0123436 (PMC4370736; doi:10.1371/journal.pone.0123436)
Supplement: S4 Table — (DOCX) [file pone.0123436.s004.docx]

| **S4 Table. Secondary antisera**  Secondary Antibodies Used | | | |
| --- | --- | --- | --- |
| **Antigen** | **Host Species** | **Dilution** | **Supplier** |
| Sheep FITC | Donkey | 1:100 | Jackson Labs |
| Rabbit FITC | Donkey | 1:200 | Jackson Labs |
| Sheep Alexa 594 | Donkey | 1:100 | Molecular Probes |
| Mouse Alexa 647 | Donkey | 1:200 | Molecular Probes |
| Mouse Alexa 594 | Donkey | 1:200 | Molecular Probes |
| Rabbit Alexa 647 | Donkey | 1:400 | Molecular Probes |
